# SARS-CoV-2-Associated Multisystem Inflammatory Syndrome in a Child in Uganda: A Paediatric Experience in a Resource-Limited Setting

**DOI:** 10.1155/2022/7811891

**Published:** 2022-02-23

**Authors:** Thereza Piloya, Lydia Nakiyingi, Ivan Kimuli, James Kayima, Joseph Lubega, John Sekabira, Hellen T. Aanyu

**Affiliations:** ^1^Makerere University, Department of Paediatrics, College of Health Sciences, Kampala, Uganda; ^2^Makerere University, Department of Internal Medicine, College of Health Sciences, Kampala, Uganda; ^3^Makerere University, Department of Physiology, College of Health Sciences, Kampala, Uganda; ^4^Baylor College of Medicine, TX, Department of Paediatric Oncology, Houston, USA; ^5^Makerere University, Department of Surgery, Kampala, Uganda; ^6^Mulago National Referral Hospital, Department of Paediatrics, Kampala, Uganda

## Abstract

SARS-CoV-2-associated Multisystem Inflammatory Syndrome in children (MIS-C) has been described in developed settings that have reported a high burden of COVID-19 cases. However, to date, there are few published cases of MIS-C that have been described in the African region. MIS-C has high morbidity and even mortality without a prompt diagnosis. We report a case of a 9-year-old girl who presented with typical clinical features of MIS-C in Uganda but had a delay in diagnosis. This case report aims to raise awareness among health providers in similar settings to improve clinical suspicion of MIS-C, facilitate prompt diagnosis and treatment, and thus improve outcomes.

## 1. Introduction

Coronavirus disease (COVID-19), caused by the novel severe acute respiratory syndrome coronavirus 2 (SARS-CoV-2) was first announced a pandemic by the World Health Organisation (WHO) on March 11, 2020. Globally, as of June 28, 2021, coronavirus cases were over 182M with approximately 4M deaths [1]. Around the same time, Uganda was experiencing a second wave of the pandemic and had cumulatively reported over 81,000 cases of COVID-19, of which 4767 (5.9%) were aged 0–19 years, with 4 deaths in this group [2]. Infections among children are usually asymptomatic or mild diseases as compared to adults as reported elsewhere [3, 4]. However, SARS-CoV-2 infection in children has recently been associated with a rare severe condition affecting multiorgans called multisystem inflammatory syndrome in children (MIS-C) (CDC-MIS-C) [5, 6]. MIS-C has been described by the CDC and WHO as a clinical entity among children infected with SARS-CoV-2 characterised by hyperinflammation with multiorgan involvement [5, 6]. The MIS-C clinical presentation has common characteristics with the presentation of Kawasaki disease (KD), including fever, high levels of inflammatory markers, and multisystem damage [7, 8].

MIS-C diagnosis can be difficult due to its diverse presentation [3, 9]. Furthermore, there is still limited knowledge and experience in the diagnosis and management of SARS-CoV-2-associated MIS-C among clinicians in a resource-limited setting (RLS). Without prompt management, MIS-C has a high risk of poor outcomes, including cardiovascular derangement, high morbidity, and mortality, therefore the need for early and proper diagnosis and treatment [10, 11]. With the small proportion of children reported with COVID-19 in most countries in Africa, clinical suspicion of MIS-C among clinicians is low, as many lack awareness, knowledge, and experience on MIS-C due to paucity of data on MIS-C from Africa. Additionally, our case had atypical presentation of MIS-C with a high risk of missed diagnosis as compared to the other cases reported in Africa [12].

We describe a 9-year-old with SARS-CoV-2 infection who presented with rapid deterioration and features of MIS-C, in whom the diagnosis of MIS-C was delayed due to limited awareness of the condition in Uganda. The report aims to raise awareness among health providers in similar settings to improve clinical suspicion of MIS-C, facilitate prompt diagnosis and treatment, and thus improve the outcome.

## 2. Case Summary

### 2.1. COVID-19 Presenting with Acute Abdomen, Mimicking Acute Appendicitis

We describe a 9-year-old Ugandan girl, previously healthy, who presented to a COVID-19 treatment unit (CTU) of a tertiary hospital as a referral from a private hospital with persistent high-grade fever which was unresponsive to antipyretics and severe abdominal pain in the periumbilical and right iliac regions for 8 days. She also had associated malaise, nonbloody profuse diarrhoea (7–10 motions/day), and vomiting which developed approximately 5 days after the onset of symptoms. She had a generalised maculopapular nonitchy skin rash that had been noticed seven days after the onset of symptoms, but this was not associated with features of conjunctivitis or reddening of the mouth. She also developed a dry, irritating cough a day prior to admission to the CTU that was associated with shortness of breath.

The child first presented to an outpatient health facility two days after the onset of the fever, weakness, and abdominal pain. She was treated with six-hourly paracetamol and an oral amoxicillin plus clavulanic acid following a complete blood count (CBC) report that showed neutrophilia. However, despite several days of treatment, the symptoms persisted with worsening abdominal pain, high-grade fevers, chills, diarrhoea, vomiting, and loss of appetite, which raised a clinical suspicion of appendicitis. An abdominal ultrasound performed at this time was essentially normal with no features to suggest an appendicitis or any other finding to explain the abdominal symptoms. Nevertheless, an empiric diagnosis of appendicitis was made and laparoscopic appendicectomy was performed. At laparoscopic inspection, the appendix was grossly normal. The child's course deteriorated postoperatively with severe lethargy and persistent symptoms despite three days of intravenous (IV) fluids, oral morphine for pain, antipyretics, and empiric broader spectrum antibiotics (piperacillin-tazobactam). The child developed photophobia. An nasopharyngeal swab for SARS-CoV-2 was performed and was found positive. At this time, approximately 7 days after the onset of the initial symptoms, the child developed a dry paroxysmal cough with shortness of breath. Her oxygen saturation (SPO_2_) dropped from 98% to 82%. A diagnosis of COVID-19 pneumonia was made, and she was transferred to the CTU at the tertiary hospital for further management.

Notably, it was established that the child was in close contact with her mother who had a positive COVID-19 PCR test on a nasopharyngeal swab about three weeks prior to her presentation at the CTU.

Examination revealed a very sick child with a maculopapular rash, oral thrush, febrile (temperature of 39.5°C), tachypneic with a respiratory rate (RR) of 35 breaths/min, SPO_2_ 80–85% on room air, and 95%–97% on 3 l/min of oxygen by nasal prongs. The chest was clear. Pulse rate was 46–70 beats/min, blood pressure was 110/60 mmHg, and heart sounds were normal. The abdominal examination revealed a laparoscopy scar, tenderness in the right iliac fossa and epigastrium with no guarding or distension, and had normal bowel sounds. She was agitated, but the neck was soft and Kernig's sign was negative.

### 2.2. Diagnostic Assessment

**Figure 1:**
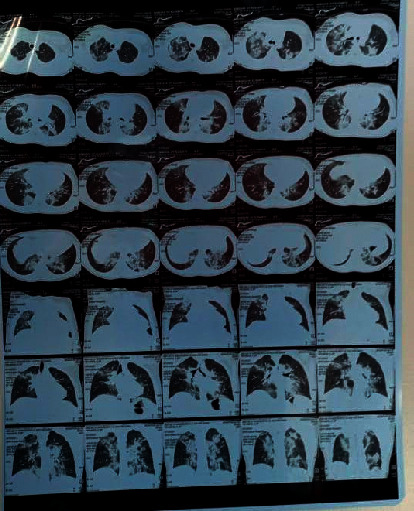
A film of the chest computerised tomography scan: features of multifocal peripheral ground glass opacification and consolidation in both lungs with basal and apical predominance. The CT severity score is 14/25 (moderate parenchymal involvement).

A comprehensive work up was performed including CBC, liver function tests (LFTs), D-dimers, C-reactive protein (CRP), ferritin, renal function tests (RFTs), electrolytes, Troponin T (results are provided in Table 1), and chest CT scan (Figure 1). Significantly, she had a lymphopenia of 720 cells/microliter (NR 1.0–7.0), a raised CRP of 103 mg/dl (NR < 1.6), and ferritin of 344 ng/ml (NR 20–250). CT scan (Figure 1) showed features of multifocal peripheral ground glass opacifications and consolidation in both lungs with basal and apical predominance.

The findings of lymphopenia, raised CRP, and ferritin and a positive SARS-CoV-2 test together with symptoms of fever and gastrointestinal tract (GIT) symptoms were consistent with a diagnosis of MIS-C [5, 6]. To investigate for Kawasaki disease, we also performed an echocardiogram and an electrocardiogram both of which were normal.

### 2.3. Treatment for SARS-CoV-2-Associated MIS-C

The child was started on intravenous immunoglobulin (IVIG) on day 10 of fever onset at a dose of 2 g/kg. She was also treated with methylprednisolone at a dose of 2 mg/kg/day as an anti-inflammatory drug and continued to receive piperacillin/tazobactam at 100 mg/kg/day for possible sepsis. Low molecular weight heparin (LMWH) was administered at 20 mg subcutaneously for 5 days as prophylaxis for deep venous thrombosis. However, the patient continued to have cardiorespiratory instability, acute kidney injury (see creatinine trend in Table 1) with oliguria of 0.6 ml/kg/hr, and central nervous system (CNS) deterioration with compensated hypovolemic shock, hypothermia of 34.5°C, photophobia, diplopia, and agitation. We attributed her CNS symptoms to side effects of IVIG. For the agitation, clonidine tablets (100 microgram) were given at night for 3 days. Her fluid and nutrition intake were maintained using a nasogastric tube feed in addition to intravenous fluids. In view of a nationwide shortage of critical care beds resulting from the COVID-19 surge, it was not possible to escalate her care to mechanical cardiorespiratory support. We continued to provide the above support in a pediatric intensive nursing setting.

### 2.4. Recovery and Follow-Up

After 72 hrs of IVIG, high-dose steroids, and intensive supportive care, the fever trended down to normal, the respiratory distress improved, and her vital organ function laboratory tests including the RFTs, LFTs, electrolytes, and inflammatory markers were trending to normal (Table 1). She was discharged home after 11 days of admission on oral prednisolone 30 mg (1 mg/kg), which was tapered over 2 weeks, aspirin 75 mg once a day for a total of 4 weeks, inhaled budesonide 200 mcg twice a day for 2 weeks, and home chest physiotherapy. Echocardiogram and electrocardiogram were repeated 2 weeks after discharge to evaluate for any complications due to the inflammation and these were normal. We plan to obtain a chest radiograph and spirometry 4 weeks after discharge to evaluate for complete resolution of lung disease and no residual lung disease.

## 3. Discussion

MIS-C associated with SARS-CoV-2 is a rare (2 in 100,000) severe clinical presentation of COVID-19 in children [9, 13]. Although it is rare, there is a need to raise awareness of this syndrome due to its diverse presentation, the emergency/critical care needed, and the possible fatal complications. We believe this is the index case reported in Uganda. Children and adolescents were less severely affected by the SARS-CoV-2 in our setting and, therefore, there was a very low index of suspicion among health workers for diagnosis of MIS-C.

MIS-C associated with SARS-CoV-2 is thought to occur secondary to a cytokine storm that damages numerous organ systems. The inflammatory response results in blood vessel dilation, leading to hypotension, fluid accumulation, and shock. It is speculated that MIS-C is a stage III-delayed immunological phenomenon associated with hyperinflammation following either symptomatic or asymptomatic COVID-19 infection [14]. It is still unknown if there is a genetic predisposition to MIS-C [14]. The reported median age of presentation is 9 years [11].

Centers for Disease Control and Prevention (CDC) defines MIS-C as a clinical condition which affects patients under 21 years of age presenting with fever >38.0°C for ≥24 hours, or report of subjective fever lasting ≥24 hours, laboratory evidence of inflammation (one or more of the following: elevated C-reactive protein (CRP), erythrocyte sedimentation rate (ESR), fibrinogen, procalcitonin, D-dimer, ferritin, lactic acid dehydrogenase (LDH), or interleukin 6 (IL-6), elevated neutrophils, reduced lymphocytes, and low albumin)), severe illness needing hospitalisation, and involvement of two or more organ systems (cardiac, renal, respiratory, hematologic, gastrointestinal, dermatologic, or neurological), with positive testing for SARS-CoV-2 indicating current or recent infection or COVID-19 exposure; and no other alternative plausible diagnoses [5]. Despite presenting with features suggestive of MIS-C as defined by the CDC, the patient was initially managed for the common illnesses in children like appendicitis and gastroenteritis. Presentation with an acute abdomen requiring surgical intervention is not uncommon in MIS-C [8, 15]. A case series of children with MIS-C from South Africa showed that children underwent laparotomy for suspected appendicitis just like ours [15]. The cause of acute abdomen is plausibly due to the hyperinflammatory state seen in COVID-19 and MIS-C, which may play a role in the pathogenesis of intestinal involvement. It is also hypothesised that there is a role of the angiotensin-converting enzyme 2 (ACE2) receptor that is expressed in the intestine [16], allowing SARS-CoV-2 to invade gastrointestinal cells.

This presentation may delay the diagnosis and lead to unnecessary surgery. Therefore, a high index of suspicion is needed to avoid a missed diagnosis. Fever and gastrointestinal symptoms like abdominal pain and nausea are overlapping symptoms for both MIS-C and appendicitis. However, the presentation of this child at the peak of the second wave of SARS-CoV-2 in our setting, with exposure to SARS-CoV-2 in the family and increased inflammatory markers should have increased our diagnostic suspicion of MIS-C. Yet, the diagnosis of MIS-C was made 10 days after the onset of symptoms in the patient, which may have led to her rapid deterioration and could possibly have led to unfavourable outcomes without a proper diagnosis.

Her specific therapy for MIS-C with IVIG and high-dose glucocorticoid was started on 10^th^ day of the onset of illness, yet it is reported that in most cases IVIG was administered between days 5 and 8 of illness [17]. We believe this would have been different if there was increased awareness among the health workers regarding MIS-C presentation.

The majority of the children with MIS-C progress into cardiovascular, and for some, respiratory dysfunction, with a reported 61% becoming hypotensive [11, 18]. Our patient showed features of circulatory shock, although she had normal BP, normal heart function on echo, and the cardiac markers were normal. However, shock and hemodynamic compromise in MIS-C can occur in the absence of laboratory evidence of myocardial inflammation and with preserved cardiac function and rapid reversibility [19]. The cause may plausibly be due to the pathogenesis of MIS-C of severe vasodilatation and even the infection. Most of the children will need inotropic support for the shock [11, 20]. However, we adequately reversed the shock in the patient with IV fluids alone, and we did not need inotropes. This could have been due to the early identification and intervention of the shock while in the hospital.

Respiratory distress which includes tachypnea, retractions, and/or increased work of breathing is common in 72% of children with MIS-C. Mechanical ventilation is required for approximately a quarter of the children with MIS-C [11]. The radiological findings vary in MIS-C, including pneumonia and/or pleural effusions identified in chest radiographs of 55.8% of children, as reported by Aronoff et al. [11], while Kaushik et al. [20] reported focal or bilateral pulmonary opacities in 33% of the patients. Subpleural ground glass opacities and consolidations with features of pneumonia have also been reported [21]. These findings were consistent with our findings of consolidation and ground glass opacities.

The patient also developed acute kidney injury as defined by raised serum creatinine for age, but this was resolved by conservative management with fluid. The AKI may have been caused by the cytokine storm of the disease, drugs used in management, or hypovolemia from the shock. Acute kidney injury has been reported in about 11.9% of children, and none of the cases reported long-term chronic kidney disease requiring dialysis [11].

Currently, specific therapy for MIS-C is based on expert opinion and previous management of hyperinflammatory conditions like KD [14]. No randomized controlled trials have been performed to date for the most appropriate therapy. Management of the MIS-C in this patient depended on previous reports [14, 22, 23] and guidance on management from the CDC and WHO [5, 6].

The goals of treatment for MIS-C are to decrease systemic inflammation and restore organ function, in order to decrease mortality and reduce the risk of long-term sequelae, such as the development of persistent cardiac dysfunction. Our case received both steroids and IVIG as recommended, with a good response clinically and in the immunological markers. Although IVIG is recommended, it is costly and would not be affordable for the majority of patients in LMICs. Furthermore, the biologic agents, interleukin-1, and interleukin-6 antagonists like anakinra and tocilizumab, respectively, are unavailable and even more costly. Therefore, if the patient needed escalation of therapy, this would not be an option in our setting. Although most of the current treatment protocols utilise intravenous immunoglobulin (IVIG) and methylprednisolone with ASA for the treatment of MIS-C [11, 23], evidence has not yet emerged regarding which regimen gives a better outcome in the management of MIS-C. A review by Mcardle et al. was inconclusive regarding evidence for superiority of any of the three treatment regimens: a combination of IVIG and glucocorticoids, IVIG alone, and glucocorticoids alone [23]. However, they found that glucocorticoids alone may reduce progression on ventilator support, and the combination of IVIG and glucocorticoids may reduce the risk of immunomodulatory treatment escalation [23]. Therefore, since IVIG and biologic agents are costly and have limited availability in many countries, more evidence is needed to support their use in preference to cheaper anti-inflammatory agents such as glucocorticoids.

MIS-C has a fairly good outcome with early diagnosis and intervention. Survival was reported at 82.2% and mortality at 1.4% [11], but intensive care treatment is needed for many children [17, 18, 20]. Even with a delay in making a diagnosis and the institution of therapy, our patient had a favourable outcome with minimal complications. We believe the outcome was good due to the absence of cardiac complications like coronary aneurysms and myocardial damage and the eventual institution of therapy with IVIG and glucocorticoids.

Additionally, evaluation of patients with evidence of MIS-C requires a multidisciplinary approach. These teams are not readily available in many facilities in LMICs, but in this case early consultations were made with the teams including intensivists, infectious disease specialists, cardiologists, pulmonologists, and pediatricians, which may have led to a good outcome.

Despite being rare, MIS-C is of significant concern due to the severity of the illness, with the majority of children requiring critical care treatment for complications to prevent unfavourable outcomes. MIS-C should be high on the differentials for patients who present with gastrointestinal symptoms and a history of recent SARS-CoV-2 exposure or infection, even if clinical findings seem consistent with other pathologies like appendicitis. Therefore, health workers in LMICs need awareness regarding MIS-C to improve outcomes.

## Figures and Tables

**Table 1:** Summary of the trend of relevant laboratory values.

Laboratory test (normal range)	Pre-admission to the CTU tertiary centre	Admission to the CTU tertiary centre	72 hours postadmission	7 days postadmission	2 weeks postdischarge
Leucocytes (K/*μ*L) (4.5–12.0)	5.5	5.41	8.47	8.3	5.75
Platelets (K/*μ*L) (140–300)	200	317	512	632	298
Neutrophils (K/*μ*L) (2–7)	2.6 (46.7%)	4.48 (82.7%)	6.72 (79.3%)	6.84 (77.4%)	3.45 (59.9)
Lymphocytes (K/*μ*L) (1.0–7.0)	2.1 (38.7)	0.72 (13.3%)	0.91 (10.7%)	1.79 (20.3%)	1.82 (31.7)
Hb (g/dl) (12–16)	11.4	11.6	11.7	11.5	11.4
Creatinine (mmol/l) (27–42)		30	77	60	45
Urea (mmol/l) (2.76–8.07)		1.5	9.7	7.2	3.6
CRP (<1.6 mg/dL)	100	103.12	9.68		2.50
Troponin (<0.3 ng/mL)			<0.1		
CPK-MB (ng/mL) (<5.0)			0.97		
Ferritin (ng/mL) (20–250)			344.5		28.44
D-dimer (up to 500 mg//ml)			492.9		
Albumin (g/dL) (2.8–4.5)		3.72		3.84	4.7
ALT (U/L) (<41)		52.3		19.2	15.9
AST (U/L) (<32)		52.3		17.9	17.2

Hb: haemoglobin; CRP: C-reactive protein; ALT: alanine aminotransferase; AST: aspartate aminotransferase; CK-MB: creatinine kinase myocardial band. Bacterial blood cultures were negative. Normal serum electrolytes (sodium, potassium, calcium, and chloride).

## Data Availability

The data regarding this case report is present at the Records Registry of the Mulago National Referral Hospital. It may not be possible to get it online due to data protection policies for patients' records.
